# Diagnosis of Endometriosis Based on Comorbidities: A Machine Learning Approach

**DOI:** 10.3390/biomedicines11113015

**Published:** 2023-11-10

**Authors:** Ulan Tore, Aibek Abilgazym, Angel Asunsolo-del-Barco, Milan Terzic, Yerden Yemenkhan, Amin Zollanvari, Antonio Sarria-Santamera

**Affiliations:** 1 School of Engineering and Digital Sciences, Nazarbayev University, Astana 010000, Kazakhstan; ulan.tore@alumni.nu.edu.kz (U.T.); aibek.abilgazym@nu.edu.kz (A.A.); 2 Department of Surgery, Medical and Social Sciences, Faculty of Medicine, University of Alcalá, 288871 Alcalá de Henares, Spain; angel.asunsolo@uah.es; 3 Department of Epidemiology and Biostatistics, Graduate School of Public Health and Health Policy, City University of New York (CUNY), New York, NY 10028, USA; 4 Ramón y Cajal Institute of Healthcare Research (IRYCIS), 28034 Madrid, Spain; 5 Department of Surgery, School of Medicine, Nazarbayev University, Astana 010000, Kazakhstan; milan.terzic@nu.edu.kz; 6 Clinical Academic Department of Women’s Health, CF “University Medical Center”, Astana 010000, Kazakhstan; 7 Department of Obstetrics, Gynecology and Reproductive Sciences, School of Medicine, University of Pittsburgh, Pittsburgh, PA 15213, USA; 8 Department of Medicine, School of Medicine, Nazarbayev University, Astana 010000, Kazakhstan; yerden.yemenkhan@nu.edu.kz; 9 Department of Biomedical Sciences, School of Medicine, Nazarbayev University, Astana 010000, Kazakhstan; antonio.sarria@nu.edu.kz

**Keywords:** endometriosis, comorbidities, machine learning, XGBoost, AdaBoost, random forest, logistic regression, decision tree, feature importance

## Abstract

Endometriosis is defined as the presence of estrogen-dependent endometrial-like tissue outside the uterine cavity. Despite extensive research, endometriosis is still an enigmatic disease and is challenging to diagnose and treat. A common clinical finding is the association of endometriosis with multiple diseases. We use a total of 627,566 clinically collected data from cases of endometriosis (0.82%) and controls (99.18%) to construct and evaluate predictive models. We develop a machine learning platform to construct diagnostic tools for endometriosis. The platform consists of logistic regression, decision tree, random forest, AdaBoost, and XGBoost for prediction, and uses Shapley Additive Explanation (SHAP) values to quantify the importance of features. In the model selection phase, the constructed XGBoost model performs better than other algorithms while achieving an area under the curve (AUC) of 0.725 on the test set during the evaluation phase, resulting in a specificity of 62.9% and a sensitivity of 68.6%. The model leads to a quite low positive predictive value of 1.5%, but a quite satisfactory negative predictive value of 99.58%. Moreover, the feature importance analysis points to age, infertility, uterine fibroids, anxiety, and allergic rhinitis as the top five most important features for predicting endometriosis. Although these results show the feasibility of using machine learning to improve the diagnosis of endometriosis, more research is required to improve the performance of predictive models for the diagnosis of endometriosis. This state of affairs is in part attributed to the complex nature of the condition and, at the same time, the administrative nature of our features. Should more informative features be used, we could possibly achieve a higher AUC for predicting endometriosis. As a result, we merely perceive the constructed predictive model as a tool to provide *auxiliary* information in clinical practice.

## 1. Introduction

Endometriosis is defined as the extrauterine growth of estrogen-dependent endometrial-like epithelial and stromal cells that are most frequently detected in the pelvic cavity, although endometriosis lesions can be found throughout the body [1]. Endometriosis is a major health issue with significant socio-economical impacts, both in terms of direct medical care and indirect costs, because of its impact on quality of life, social function, and work productivity [2]. Treatment options remain suboptimal and show significant variability in their effectiveness [3]. Moreover, there is increasing recognition that considering endometriosis exclusively as a pelvic gynecological disorder does not reflect its complex nature and diverse clinical manifestations [4].

Over the years, important advances have been made in defining the pathophysiology of endometriosis, but the cause of this condition remains ill-defined. Although pain and infertility are hallmark features of endometriosis, some people with endometriosis remain asymptomatic. Several processes may be involved in the pathogenesis of pain [5], but many aspects are still unclear [6,7]. Infertility is also typically associated with endometriosis [8,9,10], but the cause–effect connection is still under debate [11]. Diagnosis continues to present challenges. Its clinical presentation is highly variable [12]. Existing classification schemes relate poorly to its extension and severity and lack prognostic value to predict responses to treatment or disease progression [13]. The absence of specific biomarkers and the lack of sensitivity and specificity in imaging tests mean that arriving at a diagnosis of endometriosis continues to be challenging: significant diagnostic delays are related to significant dissatisfaction with medical care [14]. Given the diversity in the clinical course and the diagnostic complexities, epidemiological estimates of endometriosis are quite variable [15]; its prevalence ranges from 1 to 5%, while its incidence ranges between 1.4 and 3.5 per thousand per year [16].

Recent studies have attempted to leverage machine learning to assist in endometriosis diagnosis. In a cohort of 1734 patients with at least one symptom of endometriosis, Bendifallah et al. [17] used machine learning algorithms such as logistic regression, decision tree, random forest, and XGBoost to distinguish patients with a confirmed endometriosis status from those without a confirmed clinical examination of deep endometriosis or past treatment of endometriosis. Using 16 features related to the history, demographics characteristics, endometriosis phenotype, and treatment, they reported an area under the curve (AUC) in the range of 0.5 to 0.92, depending on the cohort and the algorithm. Another study [18] used a much larger cohort of size 148,647 to classify women with and without an endometriosis diagnosis. The study used over 1000 variables, including genetic variants (single-nucleotide polymorphisms), medical history, and variables related to lifestyle, and reported an AUC of about 0.81, which was achieved using gradient-boosting algorithms.

Endometriosis has been associated with multiple diseases [19,20], including certain cancers [21]. In-depth knowledge of comorbidities may be important to improve our understanding of this disease, contributing to clarifying its clinical complexity and variability, but also to facilitate accurate earlier diagnosis and the initiation of targeted treatments. However, most studies have explored the association of endometriosis with specific comorbidities, a perspective that does not consider the diversity of this complex disease or consider possible interrelationships between conditions [1]. The goal of this study is to investigate the possibilities of using machine learning to predict endometriosis by analyzing data obtained from electronic medical records based solely on comorbidities as well as age, unlike previous studies, where additional data, including laboratory test results, disease history, and other patient information, have been used to diagnose endometriosis. We develop a machine learning platform to construct predictive models of endometriosis. The platform uses and compares logistic regression [22], decision tree [23], random forest [24], AdaBoost [25], and XGBoost [26] for prediction, and uses Shapley Additive Explanation [27] (SHAP) values for feature (comorbidity) importance analysis.

## 2. Materials and Methods

### 2.1. Study Population

The data for this study were obtained from the Primary Care Clinical Database (PCCD), maintained by the Spanish Ministry of Health. The PCCD includes de-identified data extracted from Spanish Regional Health Services electronic medical records of their respective Primary Care Systems. For this work, the data of 627,566 women aged 15–65 years registered with their primary care centers from 2013 to 2019, with and without endometriosis, from six Spanish regions, namely, Andalusia, Basque Country, Cantabria, Catalonia, the Region of Murcia, and the Valencian Community, were analyzed. The database consists of the following information from each patient: identification codes (IDs), income, labor situation, size of residence, region, and country of origin, as well as diagnoses and medicines prescribed by primary care doctors. IDs were anonymized by the Ministry of Health. For this work, only comorbidities with a frequency of at least 5% were selected. Diagnoses were coded as a separate categorical variable that has “yes” and “no” subgroups. Diagnoses were coded using the International Classification of Primary Care, 2nd Edition (ICPC-2). For this study, a diagnosis of endometriosis was considered when a code of X99.01 was registered in the PCCD. Codes indicating a diagnosis of endometriosis are based on clinical providers’ reporting (primary care or hospital specialists). The data available do not permit us to determine whether the diagnosis is based on signs and symptoms, diagnostic images, laparoscopic visualization, or biopsy [28]. The dataset used is exempt from ethical approval, as it is a secondary anonymized dataset that is publicly available from the Spanish Ministry of Health. The data for analysis do not have any variables that permit the identification of individual patients. All methods were carried out in accordance with the “Reporting of studies conducted using observational routinely collected health data” (RECORD) guidelines.

### 2.2. Data Preprocessing

Out of 627,566 collected data, 14,789 records had one or more missing feature values. Since none of these records had a positive endometriosis diagnosis, they were simply removed from the dataset. All features that were identical across both classes (variance 0) were excluded. Stratification was used to keep the proportion of cases and controls in both the training and test sets the same as the full dataset. There were 114 comorbidities considered in this analysis as binary features and a continuous numeric feature age. Standardization was applied to the training set for normalization. Statistics obtained on the training set were used to normalize the test set.

### 2.3. Initial Model Selection

The developed machine learning platform includes five algorithms: logistic regression with L1 and L2 regularization [22], decision tree [23], random forest [24], AdaBoost [25], and XGBoost [26]. To choose these algorithms, we considered prior studies on predicting endometriosis [16,17], as well as the general view on algorithms in the machine learning community regarding their capacity to deal with tabular datasets. For example, consider the comment by J. Friedman on L. Breiman’s view on AdaBoost [29]: “In fact, Breiman (1996) (referring to a NIPS workshop) called AdaBoost with trees the “best off-the-shelf classifier in the world”. At the same time, XGBoost is considered one of the best predictive models for tabular data, and it has been widely used in competitions [30].

### 2.4. Machine Learning Platform: A General Review

**Figure 1:**
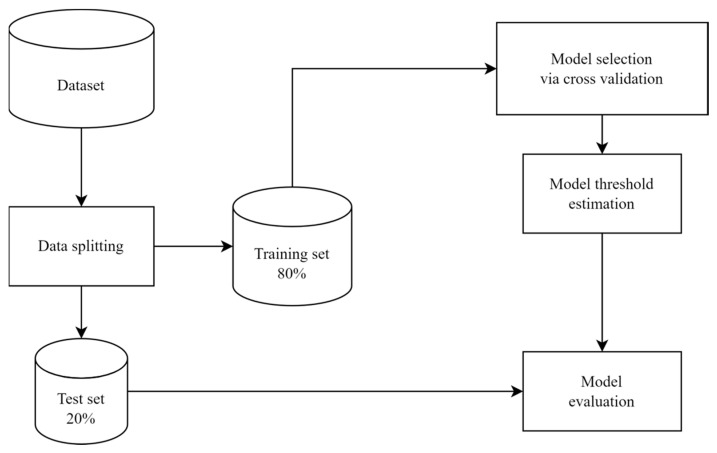
A general view of the machine learning pipeline.

Figure 1 shows the general working mechanism of the developed platform. In particular, the data were first split into training and test subsets. The training set was used for identifying the best-performing algorithm (model selection) and training our final predictive model. The best-performing algorithm was selected based on the AUC estimated using 5-fold CV. Once the best-performing model was selected, the decision-making threshold estimation was performed. Specifically, the optimal decision-making threshold for the final model was estimated based on the balanced accuracy (BA), and the model was evaluated on the held-out test set. At this stage, we report several metrics for the model: AUC, BA, sensitivity (recall), precision, and specificity, defined as:

$$sensitivity=\frac{TP}{TP+FN}$$

$$precision=\frac{TP}{TP+FP}$$

$$specificity=\frac{TN}{TN+FP}$$

$$balanced\;accuracy=\frac{sensitivity+specificity}{2}$$

where *TP*, *TN*, *FP*, and *FN* denote the number of true positives, true negatives, false positives, and false negatives, respectively.

### 2.5. Model and Feature Selection

**Figure 2:**
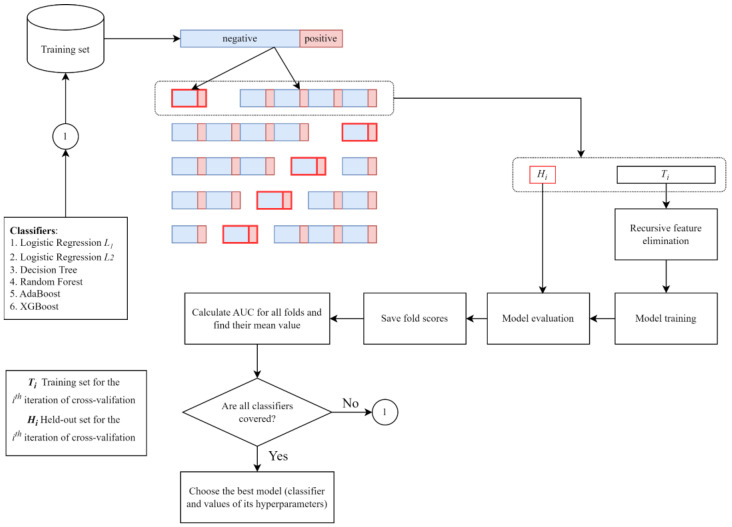
A schematic diagram of the joint model-feature selection.

We implemented a joint model-feature selection procedure to take into account the joint relationship between feature selection and model selection. For feature selection, we used recursive feature elimination (RFE), which is an embedded feature selection method [31]. We initialized the set of the number of features to select as follows: {20, 40, 60, 80, 100, 115}. For the model selection, we used grid-search cross-validation. The final joint feature subset model was selected based on the AUC estimated using 5-fold CV (Figure 2). The search space for the hyperparameter of each algorithm is presented in Table 1. The result of the joint model-feature selection is presented in Table 2.

### 2.6. Model Threshold Estimation

After selecting and fine-tuning the model, the decision threshold was identified by iterating the threshold from 0 to 1 with a step of 0.001 and computing the balanced accuracy on the training dataset. The threshold with the highest balanced accuracy on the training set was selected.

### 2.7. Model Evaluation

The selected and trained model on training data was evaluated based on the test set. In particular, the performance of the model was evaluated in terms of the AUC, balanced accuracy, sensitivity, specificity, and precision (the results are shown in Table 3).

### 2.8. Software and Packages

The computations were performed using a virtual server with an Intel Core i9 Processor, 32 GB of RAM, and 500 GB of storage, running the Windows Server 2019 Standard (64-bit) operating system. The main program was implemented in Python (version 3.10; Python Software Foundation) using open-source packages, including scikit-learn (version 1.1.2), xgboost, numpy, pandas, seaborn, matplotlib, and shap.

## 3. Results

### 3.1. Data Description

In this study, we used data maintained by the Spanish Ministry of Health for 627,566 patients with and without endometriosis (see Section 2.1). The data include 114 comorbidity (binary) features and an age (numeric) feature (Supplementary Materials, Table S1). A stratified random split was used to divide the data into training and test sets with an 80/20 ratio. This way, the test set includes 121,527 control samples and 1029 cases. The test dataset was used for evaluation model selection purposes.

### 3.2. Prediction Performance

**Figure 3:**
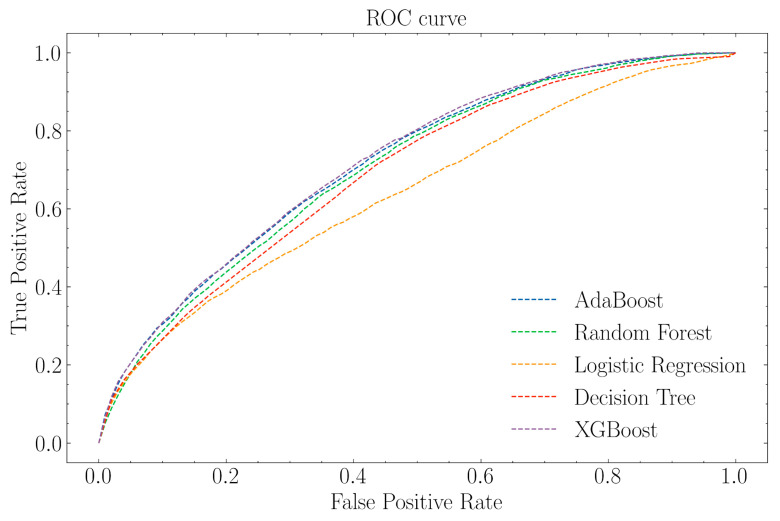
The ROC curves for the best-performing model in each class of algorithm.

The developed machine learning platform includes five classification algorithms: logistic regression [22], decision tree [23], random forest [24], AdaBoost [25], and XGBoost [26] (see Section 2.3 for the rationale behind selecting these algorithms). A pipeline consisting of feature selection and hyperparameter tuning procedures was used to train classifier models. Feature selection was implemented by applying the recursive feature elimination (RFE) technique, while hyperparameter tuning was performed using the selected features only. A grid search using 5-fold cross-validation (5-fold CV) was used for model selection. The space of hyperparameters used for each model is presented in Section 2.5. The best-performing model based on the highest AUC estimated via 5-fold CV was XGBoost (see Table 2, second column). For the selected XGBoost classifier, all 115 features were selected by the feature selection algorithm to maximize the predictive performance of the trained model. Figure 3 shows the receiver operating characteristic (ROC) curve for each of the models. Nonetheless, the AUC is independent of a priori class distribution and any specific threshold for decision-making [32]. Therefore, rather than using the “default” threshold of XGBoost for decision-making, we estimated the optimal threshold iteratively using the balanced accuracy (BA) metric. The XGBoost with the specific estimated threshold was then evaluated on the held-out test data. The results of various performance metrics obtained on test data are shown in Table 3. The confusion matrix for this XGBoost classifier is depicted in Table 4.

### 3.3. Impact Direction and Importance of Each Feature

**Figure 4:**
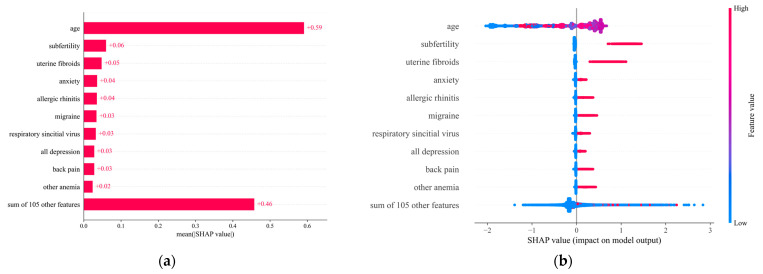
(**a**) Bar plot of top 10 features according to SHAP analysis. (**b**) Beeswarm plot of top 10 features according to SHAP analysis.

We performed a SHAP [27] analysis to (1) measure the overall importance of each feature on endometriosis prediction; and (2) infer the direction of the impact of each feature on prediction. Figure 4a and Figure 4b show the mean absolute SHAP bar plot (for ranking feature importance) and the SHAP summary dot plot, respectively, both estimated using the trained XGBoost classifier. Both figures show the top 10 features (from highest to lowest importance).

The direction of the impact is inferred from Figure 4b. Positive SHAP values for the red dots in this plot show direct dependence between the feature and the outcome, whereas the same values for the blue dots imply inverse dependence. In particular, the likelihood of endometriosis increases (decreases) for a positive (negative) SHAP value. As an example, from Figure 4a, we infer that age is the most important feature. From Figure 4b, however, we infer a direct dependence between middle-aged women and endometriosis, whereas having young or old age decreases the likelihood of having endometriosis. At the same time, we infer that infertility, the presence of uterine fibroids, anxiety, and allergic rhinitis represent other important features with a direct dependence on endometriosis.

## 4. Discussion

As can be seen in Table 3 and Table 4, the best-performing model correctly identified 76,473 true negatives (a specificity of 62.9%) and 706 true positives (a sensitivity of 68.6%). The model led to a quite low positive predictive value of 1.5%. In other words, to be able to make one correct decision (detecting one case), the model produces, on average, 66 false positives. This is not a surprising finding, given the low prevalence of endometriosis (0.8%) in this sample. However, the model rendered a quite satisfactory negative predictive value of 99.58%, meaning that women with a negative result have an extremely low probability of having endometriosis. As a result, we conclude that the developed model can be seen as a tool to provide auxiliary information for predicting endometriosis, as it can help, in combination with other diagnostic imaging tests and the clinical judgment of a specialist, to rule out a diagnosis of endometriosis. Overall, these results demonstrate the applicability of machine learning algorithms in assisting in endometriosis diagnosis.

This work complements previous works that use machine learning algorithms for screening and diagnosing endometriosis, which have focused on symptoms typically present in this condition [17,33] or biomarkers, genetic data, or diagnostic image techniques [34]. This work aimed to identify endometriosis by exploring the multidimensional association of this condition with other health problems, gynecological and not gynecological, including mental health problems [35], comparing the presence of such comorbidities in women with and without endometriosis.

The strengths of this work are the population-based characteristic of the database, which was not restricted to selected populations identified in specialized centers; the large number of cases analyzed; and the inclusion of women with and without endometriosis. Additionally, this work presents a method to diagnose endometriosis based solely on medical records of comorbidities (and age) and does not make use of diagnostic images or laboratory test results, allowing for its usage in prescreening, a typical situation in clinical settings.

Using data extracted from medical records has obvious limitations, as has been reported by other authors who have also used data from routine medical care to study endometriosis [36,37,38,39]. The data were obtained from electronic medical records based on primary care doctors’ reporting during the usual clinical management of these patients. Therefore, there may be misclassification bias as well as underdiagnosis if primary healthcare providers do not correctly include the appropriate diagnostic codes in their medical records. This problem may affect both endometriosis as well as other conditions. Relevant data, such as the extension of the diseases, the time since diagnosis, the type or severity of the conditions, and the treatments received, were not available. Diagnoses are coded based on ICPC-2. Other medical records systems code diagnoses using ICD10 codes, which are not perfectly equivalent and may return different aggregations. The data are limited to Spain, and thus, may not be generalized to other countries.

From a machine learning perspective, the separation of “model and feature selection” (Section 2.5) from “model threshold estimation” (Section 2.6) could be seen as a limitation. There were two sides to our motivation to separate these procedures: (1) The performance metric used for the joint model-feature selection is the AUC, which is independent of any specific value used as the classification threshold. Naturally, the process of estimating the best threshold for the final selected model could not be performed using the AUC metric. (2) Had a threshold-dependent metric of performance been desired and used for the model-feature selection, we could have integrated the process of threshold estimation into the model-feature selection. Nevertheless, this would have substantially increased the complexity of the implemented pipeline, which is already computationally expensive. For example, as discussed in Section 2.6, we chose candidate values of the threshold from 0 to 1 with a step of 0.001. Integrating threshold estimation into model selection with such a fine grid would increase computation a thousandfold.

## 5. Conclusions

Endometriosis is a highly diverse and complex disease. The analysis of comorbidities in endometriosis may provide a deeper insight into its multidimensionality and heterogeneity. The significant clinical variability in manifestations and responses to treatments may represent diverse plausible but presently unconfirmed pathogenesis pathways [40]. The results of this investigation are promising and showcase the potential of using machine learning to assist healthcare professionals in identifying patients with possible endometriosis and allow for more efficient screening procedures. The XGBoost classifier achieved an AUC of 0.725 on test data. At the same time, it showed sensitivity and specificity of 68.6% and 62.9%, respectively. Nonetheless, its low positive predictive value of 1.5% suggests that the model should be seen as an assisting tool, rather than the final decision maker for endometriosis diagnosis. Moreover, this study found that the top five most important features are age, infertility [41], uterine fibroids [42], anxiety [43], and allergic rhinitis [44], which are comorbidities that have been previously found with significant frequency in women with endometriosis.

## Figures and Tables

**Table 1:** Hyperparameter spaces for grid search with cross-validation model selection.

Classifier	Hyperparameter	Hyperparameter Space
Logistic Regression	Penalty	L1, L2
	Regularization parameter C	0.01, 0.1, 1, 10, 1000, 10,000, 20,000, 30,000, 40,000, 50,000, 60,000, 70,000, 80,000, 90,000, 100,000
Decision Tree	Maximum depth	1, 2, 10
	Criterion	‘gini’, ‘entropy’
	Minimum samples per leaf	1, 2, 10
	Splitter	‘random’, ‘best’
Random Forest	Maximum Features	‘auto’, ‘log2′
	Maximum depth	2, 5, 10, 20, 50, 100
	Number of estimators	10, 100, 1000, 10,000
Ada Boost	Number of estimators	10, 100, 1000
	Learning rate	0.001, 0.01, 0.1
XGBoost	Number of estimators	10, 50, 100, 200, 300, 400, 500, 600, 700, 800, 900, 1000,1250
	Learning rate	0.001, 0.01, 0.1, 1, 10, 100
	Maximum depth	5, 8, 10
	Sampling method	‘uniform’
	Gamma	0, 1, 3, 5
	Subsample ratio of columns by tree	0.3, 0.5, 0.7
	Subsample	0.7, 0.8, 0.9

**Table 2:** The AUC scores, number of selected features, and selected hyperparameter spaces of classifiers during the model selection. The best model is identified in bold.

Classifier	AUC	Number of Selected Features	Hyperparameter Space
Logistic Regression	0.646	115	Penalty: L2,
			Regularization parameter C: 8000
Decision Tree	0.693	100	Criterion: ‘gini’,
			Maximum depth: 10, Minimum samples per leaf: 1,
			Splitter: ‘best’
Random Forest	0.705	100	Maximum features: ‘auto’
			Maximum depth: 20, Number of estimators: 1000
Ada Boost	0.718	115	Learning rate: 0.1,
			Number of estimators: 1000
**XGBoost**	**0.721**	**115**	Number of estimators: 1225,
			Maximum depth: 8, Subsample ratio of columns: 0.3,
			Gamma: 1,
			Learning rate: 0.01,
			Sampling method: ‘uniform’,
			Subsample: 0.8

**Table 3:** Performance metrics estimated on the test set using the best-performing XGBoost model.

AUC	Balanced acc.	Sensitivity	Specificity	Precision
0.725	0.658	0.686	0.629	0.015

**Table 4:** The confusion matrix obtained on the test set using the best-performing XGBoost model trained on the training set: Positive and Negative labels represent patients with and without endometriosis.

		Predicted Label	
		Positive	Negative
**True label**	**Positive**	706	323
	**Negative**	45,054	76,473

## Data Availability

The data presented in this study are available on request from the corresponding author with permission from the Spanish Ministry of Health.

## Supplementary Materials

The following supporting information can be downloaded at: https://drive.google.com/file/d/1DpK3mQsT83HKG2F5LgB5RV6EDTPKgNWs/view?usp=sharing (accessed on 18 September 2023), Table S1: Features used within the study.
